# An HTML5-Based Pure Website Solution for Rapidly Viewing and Processing Large-Scale 3D Medical Volume Reconstruction on Mobile Internet

**DOI:** 10.1155/2017/4074137

**Published:** 2017-05-30

**Authors:** Liang Qiao, Xin Chen, Ye Zhang, Jingna Zhang, Yi Wu, Ying Li, Xuemei Mo, Wei Chen, Bing Xie, Mingguo Qiu

**Affiliations:** ^1^Department of Medical Image, College of Biomedical Engineering, Third Military Medical University, Chongqing, China; ^2^Department of Computer Science, College of Biomedical Engineering, Third Military Medical University, Chongqing, China; ^3^College of Software and Computer, Chongqing Institute of Engineering, Chongqing, China; ^4^Department of Digital Medicine, College of Biomedical Engineering, Third Military Medical University, Chongqing, China; ^5^Department of Radiology, Southwest Hospital, Third Military Medical University, Chongqing, China

## Abstract

This study aimed to propose a pure web-based solution to serve users to access large-scale 3D medical volume anywhere with good user experience and complete details. A novel solution of the* Master-Slave* interaction mode was proposed, which absorbed advantages of remote volume rendering and surface rendering. On server side, we designed a message-responding mechanism to listen to interactive requests from clients (*Slave* model) and to guide* Master* volume rendering. On client side, we used HTML5 to normalize user-interactive behaviors on* Slave* model and enhance the accuracy of behavior request and user-friendly experience. The results showed that more than four independent tasks (each with a data size of 249.4 MB) could be simultaneously carried out with a 100-KBps client bandwidth (extreme test); the first loading time was <12 s, and the response time of each behavior request for final high quality image remained at approximately 1 s, while the peak value of bandwidth was <50-KBps. Meanwhile, the FPS value for each client was ≥40. This solution could serve the users by rapidly accessing the application via one URL hyperlink without special software and hardware requirement in a diversified network environment and could be easily integrated into other telemedical systems seamlessly.

## 1. Introduction

The 3D visualized reconstruction, which is built from sectional medical images (SMIs), such as CT, has an advantage of directly displaying the focus of a disease and is widely used in many fields, including diagnoses, case discussion, education, and patient consultation [1, 2]. In the era of mobile Internet (WIFI or 3G as fundamental carrier), with increasingly large amounts of SMI data of high quality, people desire to rapidly view and process this 3D visualized reconstruction of high quality in a diversified network environment via different client devices without installing special software [3]. In particular, many studies have reported that there were no significant differences of diagnostic accuracy regarding more and more diseases between mobile devices and the traditional workstations that promoted the further development of medical imaging technology on mobile Internet [4–9]. Presently, the 3D visualized reconstruction techniques in medicine have fallen into the following two main categories: surface rendering and volume rendering [10], both with their own characteristics to adapt to the Internet application.

Surface rendering in medicine extracts the interested surface contour of the anatomical representation from SMIs into a 3D geometrical model, which is usually stored as a VRML, VTK, or X3D file [1, 11, 12]. Some Web3D technologies, such as WebGL, could directly support these models via most of the major web browsers [13] with a very good native user experience and that is a popular way to utilize Internet applications for medical 3D visualization. However, because the information integrity depends on presetting the interested area, the client user cannot arbitrarily view the internal anatomy structure or analyze the complex organization relationship. Therefore, surface rendering technology has been mainly used for special disease with specific requirements [11, 12, 14], and it could not adapt to the custom behavior of the client user in mobile Internet very well.

Volume rendering describes a wide range of techniques to generate images from 3D scalar data [15]. It is very convenient to view the internal anatomy structure and describe the complex organizational relationship through user adjustment of the parameters of color and opacity on each voxel [16]. However, volume rendering requires a complete computation of the original volumetric data for each user's behavior event, the cost of computation being much higher than that for surface rendering. Thus, volume rendering technology is often deployed on a dedicated image workstation and in a private network environment, where the scope of application is limited. Some low-level graphics APIs, such as OpenGL ES 2.0, have been integrated into WebGL for Internet application, which could be theoretically enabled to support GPU-based direct volume ray-casting implementations, or support some slice-based sampling techniques to get close to the imaging result of ray-casting-based implementations. Nevertheless, the imaging quality and efficiency is very limited, not just because of performance of common GPU, but also because of the limited performance of web pages [7–9]. Furthermore, there is still high requirement to network for original volumetric data transmission. Currently, there is a remote volume rendering scheme to solve the limit. This scheme deploys the volume rendering job on the server side to a real-time response to behavior instructions from the client and transmits the 2D projection image from volume rendering to the client display, where the client is only an interface for sending instructions and displaying results [9, 17–20]. For example, the user slides the mouse from point A to point B to express an operation of 3D rotation on the client side, and the server just calculates the final viewing angle from A to B and sends the final result to the user. Because there is no original data downloading and no rendering cost on the client side, the network load and computing pressure of the client could be significantly reduced. However, this interaction model of “the client sends one operation request, the server replies one result at a time” may cause a bad user experience. Specifically, it may lose the value of Frames Per Second (FPS), one of the most important parameters of the 3D interactive experience. Therefore, all of [9, 17–20] discretized the trajectory of one single operation request from the client into several request points to request continuous responses from the server. For example, if the path between mouse points A and B were converted into several separated remote volume rendering requests from the client, the sever would respond to each request and send continuous 2D projection images to the client. Thus, the value of FPS could be increased; however, it may lead to a huge pressure on the server load (response to a large amount of requests) and network load (continuous data stream). Additionally, [9, 17–19] ask the client to install customized software or plug-ins, which may lead to a lack of compatibility. Therefore, existing remote volume rendering methods do not adapt to the mobile Internet application characteristics of lightweight [21] very well, which ask for lower resources of network and computing, and avoid unnecessary installation.

In this study, we plan to combine the advantage of surface rendering technology based on WebGL with the advantage of remote volume rendering to design a solution for viewing and processing 3D medical volume reconstruction in a pure webpage environment. Finally, a visualization interaction platform would be built. This platform could be used anywhere, in a diversified network environment and diversified client devices, with a good user experience similar to the native application.

## 2. Computational Methods and Theory

The core concept includes the Slave model and Master volume. The Slave model is a contour model rendered by surface rendering technology, running on WebGL as a 3D interactive interface on the client side. The* Master* volume is a volumetric data rendering from original SMIs on the server side. The final required image of high quality is a projection drawing from the* Master* volume according to the behavior instructions from the* Slave* model. This solution could be named the* Master-Slave* dual-channel interaction mode and is expected to enhance the user experience similar to the native application in a pure web page, to reduce the pressure of the server load and network load, and to meet the requirement of lightweight volumetric data sharing in a mobile Internet application.

### 2.1. Design of the* Master-Slave *Dual-Channel Interaction Mode

The architecture of this solution constitutes the following two parts: the server side and client side. The server side renders the* Master *volume to generate the final required image of high quality, while the client side provides the* Slave* model's interactive navigation.

**Figure 1:**
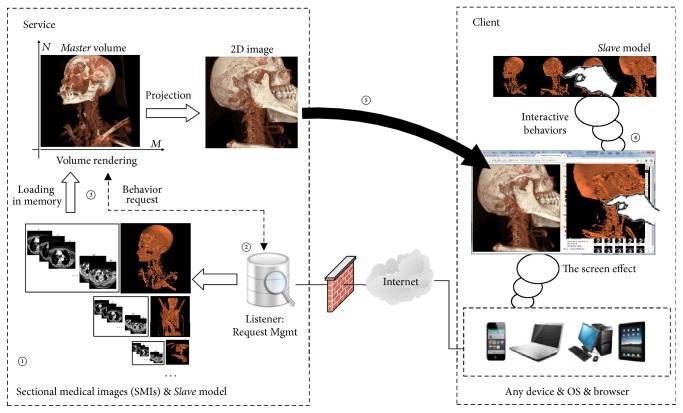
Architecture of the pure web-based solution via the* Master-Slave *dual-channel interaction mode.

The detailed procedures are as follows (schematic diagram is shown in Figure 1):

On the server side, in zone ①, groups of SMIs could be uploaded to the server through the network interface of PACS or removable storage medium. Thereafter, the relevant* Slave* models could be automatically pregenerated by rough contour extraction. The listener in zone ② accepts the user's web behavior request (instructions) in real-time and guides zone ③ to load and render the relevant SMIs in memory, which is defined as the* Master* volume, generating a 2D projection image from the rendered result for client downloading.

On the client side, while the user wants to view and process a group of SMIs, the client could download the corresponding* Slave* model from the server and reconstruct it locally in zone ④; meanwhile, request the server to load and render relevant SMIs in memory. Thereafter, in zone ④, the user could view and process the local* Slave* model arbitrarily and submit the final behavior request (instructions) to the server to acquire the required projection image from the* Master *volume in zone ②  ③  ⑤.

Here, the client could be a pure web browser running on diversified devices, supporting the WebGL technique. That means there is no installation for the client.

### 2.2. Rendering of the* Master* Volume

**Figure 2:**
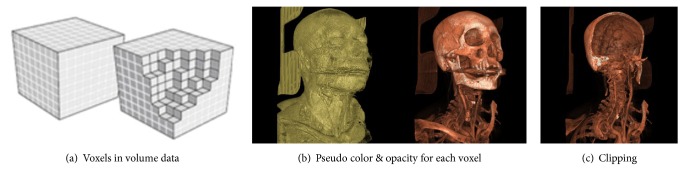
Schematic diagram of the direct volume rendering method.

We adopted the ray-casting algorithm [22] to reconstruct the* Master* volume of SMIs. The algorithm is a direct volume rendering method that has been widely used for radiodiagnosis but requires a high computing performance of the device. First, we transformed the SMIs into volume voxels in 3D space (Figure 2(a)) and then simulated rays to cast each voxel with a customized color and opacity [23] into 2D space (Figure 2(b)). This method could be used to describe a complex organization relationship. Furthermore, it could display internal anatomy structure via removing (clipping) part of the voxels that simulate a user's behavior of dissection (Figure 2(c)).

Here, we directly used the VTK (Visualization Toolkit) library [24] to realize the ray-casting algorithm. For customized color and opacity, several frequently used schemes could be deployed on the server side in advance; a dropdown list was designed on the user interface to correspond to each scheme for remote volume rendering [25, 26]. For clipping, VTK provides a class of vtkPlane to build a virtual Clipping plane to guide the voxel removal [27]. It means the clipping job only needs the specific location of the virtual Clipping plane in the 3D coordinate system. The specific location could be described by the following two parameters in the computer graphics method:* normal vector* from the origin point to the plane and the* coordinate position of a point* on the Clipping plane, both from the client submission.

### 2.3. Generation and Interaction of the* Slave* Model

The* Slave* model was pregenerated on the server side and was designed to be a navigational tool to interact with the Web page. While clients want to view and process the* Master* volume, the* Slave* model could be downloaded to the client and reconstructed on the client side.

#### 2.3.1. Generation and Slimming of the* Slave* Model

**Figure 3:**
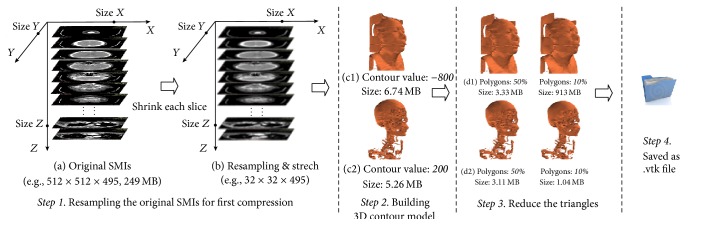
Schematic diagram of the generation and compression of the* Slave* model.

To interact with mobile Internet, the data size of the model should be as small as possible (much slimmer), while the clarity of the contour is acceptable. However, using the traditional compression-decompression algorithm may ask the client to install additional software. Hence, the steps in the generation of a smaller* Slave* model can be designed as follows (Figure 3). Here, we used a group of SMIs of the head and neck CT as an example, and the resolution of voxels is 512 × 512 × 495, 249 MB (testing data (1) in Section 4).

**Step 1 (resampling the original SMIs for first slimming).**

Load the corresponding SMIs into memory; shrink each sectional image to a standard size of 32*∗*32 via the resampling of the 2D space (Figures 3(a) and 3(b)).

**Step 2 (building the 3D geometrical contour model).**

A marching cubes algorithm [28], one of the most common methods of surface rendering, was used to extract the interested surface contour of the anatomical representation from resampling SMIs into a 3D geometrical contour model. Here, according to the feature of the CT grayscale [29], we set a particular contour value at −800 or 200 that could separate the skin or skeleton of the body from the resampling SMIs, respectively, to acquire a skin model or skeleton model (Figures 3(c1) and 3(c2)). In this case, the data size of the skin model and skeleton model could be reduced to 6.74 MB and 5.26 MB, respectively.

**Step 3 (reducing the total number of polygons for second slimming).**

The 3D geometrical contour model from Step 2 is constitutive of polygons, and we could reduce the total number of polygons to further reduce the data size. In this case, the number was reduced to 50% and 10%, and the data sizes were reduced to approximately 3 MB and 1 MB, respectively (Figures 3(d1) and 3(d2)).

**Step 4 (saved as .*vtk* model file).**

The slimmed model would be saved in a file of the  .*vtk* format, which is a storage format of the geometrical model supported by WebGL, and then stored in the same directory with original SMIs.

The result of Figures 3(d1) and 3(d2) preliminarily showed that the* Slave* model whose data size is approximately 1 MB still could be fit for interactive navigation (more experiments detailed in Section 4.1). Moreover, an additional mutual interface of the client was designed as a remote parameter setting of the contour value and the total number of polygons, to permit users to customize the* Slave* model as desired.

#### 2.3.2. Design and Quantization of the Interactive Behaviors in the* Slave* Model

For a friendly interactive experience on the client side, a simple style of interactive behaviors of the client and corresponding quantitative methods should be designed to guide remote volume rendering, including the* viewing angle request* and* clipping request*.

**Figure 4:**
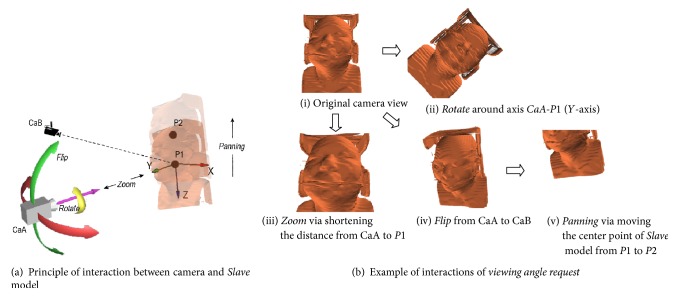
Principle of the interaction of the* viewing angle* between the camera and* Slave* model and the corresponding example.

*For the Viewing Angle Request*. The interaction of* viewing angle* includes rotation, flipping, zooming, and panning [30]. We defined the interactive 3D scene including only one* Slave* model and one camera and customized an interactive mode of the camera in VR technology to meet our requirement.  Figure 4(a) shows the principle of the interaction of the* viewing angle* between the camera and* Slave* model. We limited the lens of the camera to always be taken on point *P*1  (0,0, 0), which was the origin of the world coordinate system. The interactive behaviors of the* viewing angle* were carried out by only changing the camera position except panning. For panning, it was carried out by changing the* Slave* model position; see examples in Figure 4(b).

According to the definition, while sending request instructions to the server for remote volume rendering, the instructions only include three quantization parameters from the client to accurately describe the interactive behavior of the* viewing angle request* to the* Slave* model. The three quantization parameters include the following: attribute* view up direction*, attribute* position* of camera, and attribute* position* of the center point of the* Slave* model.

Here, attribute* view up direction* of camera indicates the direction of the top of the camera. For example, (0, −1,0), a value of the* View Up direction*, indicates the camera is perpendicular to the* XZ* plane in the world coordinate system, and the top of the camera is in the opposite direction along the *Z*-axis (see the relationship between camera CaA and* Slave* model in Figure 4(a), and corresponding example (i)* original camera view* in Figure 4(b)).

In fact, WebGL has provided some interaction methods of the moving camera to view 3D objects via mouse- and gesture-based interactive behaviors, which were packaged into a library of TrackballControls.js for a web page script. We could directly utilize the js library to acquire the three quantization parameters. However, we need to rewrite the js library to restrict and customize the interactive mode of the camera that we have defined, to ensure the consistency of the interactive rule between the* Master* and* Slave* side.

**Figure 5:**
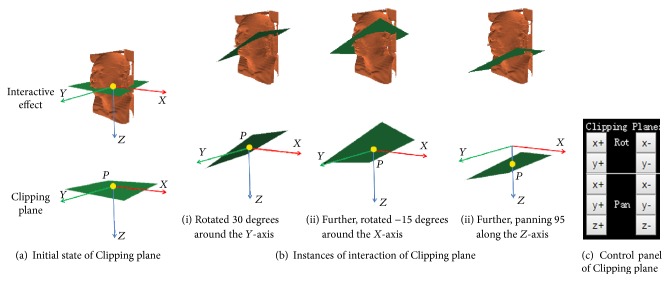
Design of the interaction of clipping to the* Slave* model and corresponding example.

*For the Clipping Request*. For arbitrarily clipping and viewing the internal anatomy structure of the* Master* volume, a green rectangle object, named the Clipping plane, was added in the above defined interactive 3D scene, to allow users to mimic the clipping behavior in the* Slave* model; see Figures 5(a) and 5(b). Here, a virtual control panel was designed separately to support the interactive behaviors of users using the Clipping plane (Figure 5(c)).

During the initialization phase, the Clipping plane covers the *XY* plane in the world coordinate system (Figure 5(a)). Its specific location could be accurately described by the following two quantization parameters in the computer graphics method:* normal vector* from the origin point to the Clipping plane, and the* coordinate position of a point* on the Clipping plane. Here, its default* normal vector* was {0,0, 1}; one* point P* on the plane was on the position (0,0, 0).

For intuitive operation of the user experience, the interactive behaviors regarding the Clipping plane include rotation and panning, and both can be operated by a virtual control panel. However, for the accurate description of the request instructions for remote volume rendering, the two quantization parameters should be used.

Therefore, formula (1) was deduced to calculate the* normal vector* from the angles of rotation.

(1) normal  vector=sin⁡θy,−sin⁡θx·cos⁡θy,cos⁡θx·cos⁡θy.

Here *θ*_*x*_, *θ*_*y*_ are the final rotation angles that rotate the Clipping plane clockwise around the *X*-axis and *Y*-axis, respectively.

For example, in Figure 5(b), a user rotated the Clipping plane 30 degrees clockwise around the *Y*-axis, and −15 degrees clockwise around the *X*-axis, and then panned the Clipping plane 95 pixels in the positive direction along the *Z*-axis. While submitting for remote volume rendering, *θ*_*x*_ = −15, *θ*_*y*_ = 30, the* normal vector* was {0.5,0.2241,0.8365} according to formula (1). Meanwhile, according to the characteristic of monolithic translation, the one* point P* on the Clipping plane was moved to* position *(0,0, 95). That would be the two quantization parameters of request instructions for clipping.

### 2.4. Remote Interaction between the Server and Client

The interactive behaviors of the* Slave* model include* viewing angle request* and* clipping request*. Moreover,* color/opacity request* is operated by the dropdown list of the preset schemes (detailed in Section 2.2). All of these requests would be submitted to the server in the form of quantization parameters, which are named as request instructions.

However, in addition to responding to the client's instructions, the server has to find an efficient way to manage and allocate system resources, to enhance the robustness of the high frequency of interaction among multiple users, for example, to reduce the probability of deadlock, cumulative delay, and task-pipeline crossing.

In our system, we used relational database schema to manage the request instructions from the client and to allocate the system resources for guiding 3D rendering. The core tables and their data storage structure are designed in Table 1.

According to Table 1, if a user chooses a group of SMIs from a list, derived from the* base table*, the server would query the* pipeline table* whether the SMIs is serving the client. If No, the SMIs would be loaded in memory, and a task-pipeline between the user and SMIs would be built. Thereafter, the* state *of the* pipeline table* would be changed to “Yes.” While being Yes, the client would be permitted to make the next interactive request. After that, for each request from the client, the server must query the* state* of the* behavior table* whether the pipeline is “pending” or “processing.” If No, the server would accept the request with a record of a “pending”* state*. Additionally, the listener on the server would real-time arrange these pending requests to the server rendering in queue. While a request is being rendered, the corresponding* state* of* behavior table* would be changed as “processing” to prevent malignant submission. While the job is finished, the* state* would be changed to “finished,” and a 2D projection image would be generated and saved in the specified HTTP sharing path.

The client adopted AJAX (Asynchronous JavaScript and XML) [31] to dynamically query the* state* of* behavior table* from the server at a fixed frequency of 300 ms. While rendering was finished, the client would download and update the 2D projection image via the HTML + js web page technique.

## 3. System Description

### 3.1. System Architecture

While Figure 1 depicts the* Master-Slave* dual-channel interaction mode, it also presents the overall architecture of the final platform. Here, we adopted the browser/server architecture, for which the communication with each other was via the HTTP protocol. The server side consists of the* Listener *&* Request Management Server *(in zone ②) and* Rendering Server* (in zone ③). The former accepts and manages the requests from the client and guides the* Rendering Server* to an orderly response. The latter renders the SMIs and generates a 2D projection image for the client downloading via HTTP. At the same time, the client side was designed by the standard of HTML5 [32]. We used <img>, an HTML image tag, to display the 2D projection image in the form of pseudo 3D, and used AJAX + js technology to realize flicker-free page updates and client RIA (rich Internet application) over diversified web browsers without installation. Moreover, HTML5 includes the WebGL standard, and the* Slave* model could run on any HTML5-supporting web browsers without any web plug-ins or special software tools. Thus, the whole platform system could run under mature network technologies.

### 3.2. System Front-End

**Figure 6:**
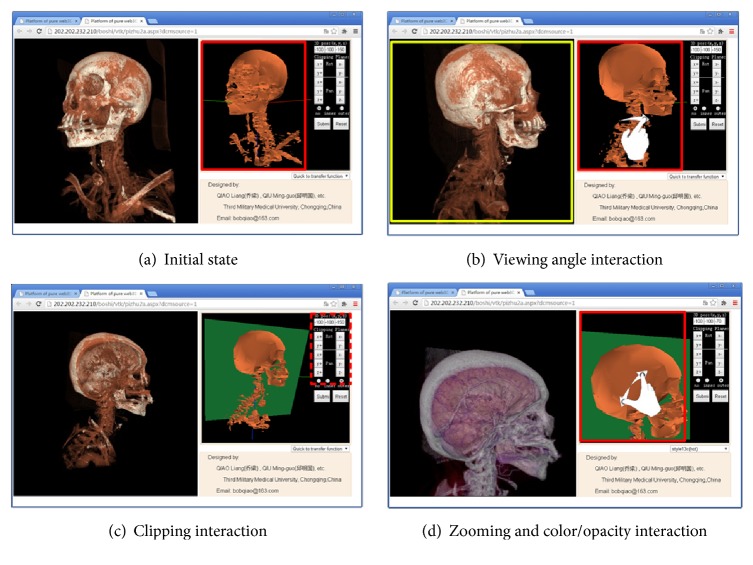
Screenshot and operational demonstration.

System front-end is an HTML5-based pure website that provides entrance to SMI access and 3D presentation. The users require a username and password to enter the system, or any experts could be invited to enter a specific task-pipeline with one URL hyperlink from a help seeker. In brief, using an HTML5-supporting web browser, including the major versions of Firefox, chrome, Opera, safari, IE 10+, the 3D volume can be viewed and processed as in Figure 6 and the Supplementary Materials available online at https://doi.org/10.1155/2017/4074137 (1. Demonstration of the operation procedure on laptop via website.mp4).

Here, the interactive behaviors of the user include the* viewing angle*,* clipping,* and* color/opacity* transformation. The first two items were operated on the* Slave* model, which was reconstructed on the client side. While the user wants to observe the final required image of high quality, the user just presses the submit button to submit the current behavior request instruction to the server and waits for the final 2D projection image to be updated.

Figure 6(a) presents the initial state of the* Slave* model (right) and 2D projection image (left). In Figure 6(b), mouse or hand gesture is used to operate the* Slave* model to transform the* viewing angle* from Figure 6(a) to Figure 6(b) (red solid box), and then the submit button is pressed to send the behavior request instruction to the server to require an updated projection image from the* Master* volume (yellow solid box). In Figure 6(c), using virtual control panel (red dashed box) to operate Clipping plane, after submitting, the new projection image was then updated (left). In Figure 6(d), the mouse wheel or hand gesture is used to zoom the* Slave* model, and then the color/opacity theme is submitted via the dropdown list (bottom right corner) to guide remote volume rendering.

## 4. Mode of Availability of the System

In this paper, we designed a* Master-Slave *dual-channel interaction mode to improve a problem of remote visualization interaction of the volume reconstruction of SMIs, which usually have a huge data size and calculation cost. Finally, a visualization interaction platform has been built. Particularly, there is no special software and hardware requirement for the clients, and no special network environment is needed.

To measure whether the effectiveness and performance to adapt to the mobile Internet application has characteristics of lightweight, three radiologists with more than 3 years of experience were invited to test the interactive experience, imaging quality, and compatibility over diversified Internet devices. Moreover, four junior students majoring in Biomedical Engineering were invited to test and quantize the network load and response time.

*Testing Data*. 3 groups of SMIs from the radiology department of Southwest Hospital of Chongqing include the following: (1) head and neck CT with 495 slices, 512*∗*512, 249.4 MB, (2) trunk CT with 609 slices, 512*∗*512, 306.5 MB, and (3) thorax and mandible CT with 500 slices, 512*∗*512, 251.6 MB.

*Server*. An IBM X3650M4 workstation (CPU: 4 × E5-2603, RAM: 16 GB, OS: Windows Server 2008) was connected to the Internet via 10 Mbps. The 2D projection image was set as the resolution of 512*∗*512 with high quality JPEG compression (level-8).

**Figure 7:**
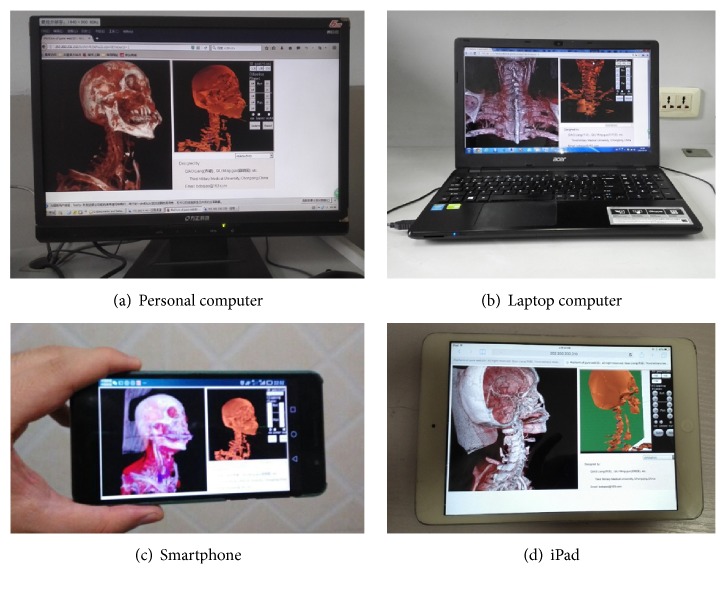
The system is accessed via four different devices and web browsers under extreme bandwidth.

*Client*. 4 Internet devices were located at the 3G or WIFI mobile Internet with the server. In Figure 7(a), there is a Founder E520 personal computer running on winXP with Firefox 46 web browser via 3G usb card. In Figure 7(b), there is an Acer E15 laptop running on win7 with chrome 43 web browser at WIFI. In Figure 7(c), there is a HuaWei Honor 6 smart phone running on EMUI 3.0, which is developed from Android 4.4.2 with the Opera 37 web browser at 3G net. In Figure 7(d), there is an iPad mini running on IOS6 with safari at WIFI. All of them could be representative in the mobile Internet. Additionally, each was limited to a 100-KBps wireless bandwidth by 360 security firewall for extreme test [33].

### 4.1. Interactive Experience, Imaging Quality, and Compatibility

To ensure the consistency of the context, the testing data (1) were considered as the main object of reference. Here, the demonstration and effect of the testing data (1) are shown in Figure 6.

Figure 6 shows that this* Slave* model of the testing data (1), which was compressed to 1.04 MB, could be fit to the demands of interactive navigation to accurately guide remote volume rendering, and shows that the projection image could be displayed to keep the original quality.

For further validation, three radiologists were invited to test the platform with the similar behaviors in Figure 6. This job was repeated to operate three groups of different testing data through different web browsers and Internet devices that were all aforementioned. Additionally, a five-point scale was designed to respond to the radiologists' requests (Table 2).

Table 2 presents a very positive evaluation from the statistical result of the imaging quality and interactive experience, and all three radiologists expressed that is an interesting way, whether as a gesture-based or mouse-based interaction. No installations were required from the client, and good compatibility was obtained (same result is shown in Table 3). However, one radiologist thought the default* Slave* model of the testing data (1), with a skeleton model of 1.04 MB (Figure 3(d2)), was a little rough. He suggested to use the skeleton model of 5.26 MB or skin model of 913 KB (Figures 3(c1) and 3(c2)) instead of the 1.04 model, both of which are the alternatives from the mutual interface for the client, as detailed in Section 2.3.1. Additionally, all three radiologists claimed that the response time for final high quality image is always about 1 second that is consistent with the test result in Section 4.2. However, they did accept the performance, because they could directly operate the* Slave* model smoothly without any delay on client side; thus a short wait for final high quality image had little influence. Moreover, one of the radiologists specifically expressed the native application of 3D rendering on desktop PC also needs 1 or 2 seconds' delay for final image; this performance of the solution running on smart phone is similar to the native application on PC and is very good.

### 4.2. Response Time and Network Load between the Client and Server

The response time can be defined as the time interval between the user sending a behavior request to the system and web browser loading the response results completely. The network load can be defined as the amount of data transmission of each interactive behavior. Four junior students majoring in Biomedical Engineering were invited to simultaneously test the platform for one hour. During the test, all of the students, respectively, viewed and processed the testing data (1) through the platform and performed similar behaviors to those detailed in Figure 6. This test was repeated by each student with different web browsers and Internet devices, all of which were aforementioned. Here, we trained the students to record the real-time network traffic by the 360 security firewall and the time interval by the script log. The results are showed in Table 3.

In Table 3, for each device, the amount of data transmission and response time of the* first loading *are much higher than the* behavior request* and* idle status* because the* first loading* asks the client to download the necessary* Slave* model. The response time of the* first loading* is <12 s at 100-KBps client bandwidth, which is similar to the total time spent on* Slave* model downloading. In fact, the response time of* first loading* also includes the cost time of SMI loading and rendering on the server side, but the server monitor presents the cost time as <7 s because SMI loading and* Slave* model downloading are simultaneous, and this cost could be neglected.

After the first loading, the network load of the* behavior request* for final image is spent on request instructions submission, final projection image downloading, and the server processing status querying at a fixed frequency of 300 ms, where the projection image downloading is the main part. The results present a complete amount of data transmission of each behavior request is close to 50 KB, but the corresponding response time for final image is approximately 1 s in 100 KBps. That is because, besides the network load, the rest of time is spent on server processing and time interval of querying (300 ms). The short delay could be acceptable for practical applications, as detailed in Section 4.1.

While in the* idle status*, including the user's interactive behavior (operation) on the* Slave* model, the bandwidth occupation is almost negligible. Moreover, there are no significant differences among diversified devices and networks at 100-KBps client bandwidth.

Additionally, the FPS of* Slave* model rendering on the client side is measured via stats.min.js, which is a library packaged from WebGL. When the client rendering is completed, FPS could remain at the upper limit.

## 5. Discussion and Comparison

Due to the nonprivate network environment and diversified purposes, application software on one device must compete for limited resources of the network and computing. Thus lightweight, which requires lower resources of the network and computing and avoids unnecessary installation, could be an important characteristic of mobile Internet application. Additionally, to avoid compatibility problems caused by different operating systems and client hardware characteristics, using HTML5 and related web technologies running on major web browsers may be a choice to adapt to cross-platform deployment and the characteristics of lightweight [21].

Therefore, this article described research on a method for the* Master-Slave *dual-channel interaction mode, which is based on the HTML5 standard (also a wider web compatibility standard of WebGL), to permit users viewing and processing million-megabyte-class SMIs via different client devices (without hardware or operating system constraints), over a pure web page (without installing special software) and in a diversified network environment.

Compared with the traditional PACS mode of “image compression, transmission, local reconstruction,” because our method does not transfer original SMIs to the client, it neither needs to wait for a long time to download nor requests special demands of the software environment on the client device. Moreover, compared with the state of the art of local volume rendering based on WebGL and HTML5 technology [7–9], our methods have no special demands of client hardware for computation that may reduce battery consumption [9] and enhance the compatibility of the client devices; our methods may be more fit for mobile Internet application. Additionally, the client does not access the original SMIs, which could be much safer.

Compared with popular solutions based on absolute surface rendering technology [11, 12, 14], our method has locally used this technique to only support light* Slave* model for interactive navigation; therefore, the demands of the network bandwidth and device rendering capability are almost negligible in our method. Moreover, the final required image of high quality is a projection drawing from volume rendering technology, indicating that our method could be convenient for users to view the internal anatomy structure and complex organization relationship; however, surface rendering technology could not support these characteristics of volume.

Compared with solutions based on absolute remote volume rendering methods, our method adopted the* Master-Slave *dual-channel interaction mode to strengthen the user experience, which is an entirely different approach from the mode of “continuous request, continuous response” of studies of [9]. Although studies of [17–19] proposed several methods to improve the mode to reduce the network load, including video-compressed transmission [17], tile-based transmission [18], and variable resolution transmission [19], the pressure of server load and real-time data stream increased. We used the same SMI data (testing data (1)), the same quality of final required image, and the same server configuration and volume rendering algorithm in Section 4 to test the methods in [17–19]. For quantitative test, we formulated an interactive trajectory with fixed 30 request points, theoretically, assuming 30 rendering requests triggered per 1 second; there should be 30 fps render effects. On this basis, [17] adopted 512*∗*512 mpeg-4 compression for transmission, [18] adopted 8*∗*8 blocks to segment each 512*∗*512 frame and transfer the blocks which are different from previous frame in the process of interaction, and [19] adopted 64*∗*64 resolution transmission in the process of interaction, but 512*∗*512 for end frame. The test was repeated for 5 times under the condition of one single client monopolizing the bandwidth and server under ideal WIFI environment. The results showed that the total amount of data transmission for each interaction test of [17–19] was 298.0 KB, 603.6 KB, and 371.3 KB, respectively, the average response time of first frame was 1.10 seconds, 0.59 seconds, and 0.52 seconds, and the average cumulative response time of end frame (30th frame which is the final required image) was 21.31 seconds, 17.61 seconds, and 16.01 seconds. The response time contains the factors of server continuous rendering response, server continuous frame-encoding, and so forth (more than 70% server CPU utilization during interaction), but the network factors could be tentatively neglected under the ideal single-user WIFI environment. Assuming that we can improve the server's processing ability to completely eliminate the server response delay (perfect 30 fps interaction effect), the theoretical value of the bandwidth could be 298.0 KBps, 603.6 KBps, and 371.3 KBps, respectively. In summary, the FPS value, one of the most important parameters of the 3D interactive experience, of the studies of  [17–19] was correlated reciprocally with the number of simultaneous tasks on the server side (server task-pipelines), server processing ability, and network bandwidth, and an obvious delay of interaction was clearly presented. By contrast, all of the Internet devices chosen to test our method could provide an FPS value at 30–60, and the FPS value was not correlated with server tasks, server processing ability, and network bandwidth, but with the device GPU (Graphics Processing Unit). Undoubtedly, almost all GPUs of Internet devices in the market could meet the demands of our light* Slave* model rendering. Moreover, the peak value of the bandwidth under the behavior request for final image was less than 50 KBps and less than 1 KBps at idle (including the operation of* Slave* model); thus, both were far below those tests of methods of [17–19]. Thus, our method could be more suitable for a complex mobile Internet environment. Moreover, the complex compression transmission methods of [17–19] were built on customized software of plug-ins for the client, which is hard to apply to pure web pages and falls short of wide compatibility. By contrast, that is our method's advantage.

For the server load, because the* Master* volume in our method only responds to the final behavior request (instructions) instead of the continuous request of the studies of [9, 17–19], the test showed that server CPU utilization was less than 20% at idle (including the operation of* Slave* model), and less than 30% for responding to behavior request; thus, it was far below of those tests of methods of [17–19] with nearly a full load during all the operation time under the same conditions of SMI data, quality of final required image, server configuration, and volume rendering algorithm. Theoretically, our platform can respond to more independent tasks (pipelines) at the same time. Table 2 shows that while the server was running four independent tasks simultaneously, the response time of our method remained at approximately 1 s in the 100-KBps client bandwidth, much better than [17–19], which was with more than 16 seconds' delay of end frame in the single task status. In fact because user viewing and processing of the 3D medical volume occurred via interaction on the* Slave* model, the delay experience was much fewer than that in [17–19], as detailed in Section 4.1. That indicates our method could have some more advantages, including much less network load and server load, to serve much more independent tasks and users.

In addition, [17–19] did not propose an effective interactive method to clip the 3D volume, what our method has accomplished.

## 6. Conclusion

This paper proposed a* Master-Slave* dual-channel interaction mode, which absorbed the advantages of the surface rendering of WebGL and remote volume rendering, to build a visualization interaction platform to rapidly view and process 3D medical volume reconstruction in a pure webpage environment. This platform could be used in a diversified network environment (100-KBps bandwidth for extreme) and diversified client devices (no special hardware and operating system constraints), without any special installation but with a good user experience similar to native application. These features could serve authorized users to conveniently access SMIs and present 3D visualization anywhere and could be a technological basis of online communication among doctors and patients [34]. Or, at least, it could help clinicians to arbitrarily acquire 3D image according to their clinical needs instead of the static pictures that radiologists drafted. For example, clinicians could conveniently observe the complicated relationship of skull base bone and blood vessel via this platform (Figure 6(d)); in the past, this piece of information was provided by a few static images generated from radiologist's understanding.

Additionally, this solution is designed to be followed as an HTML5 standard interface, indicating any authorized user could rapidly access the application via one URL hyperlink, and the platform could be easily integrated into other telemedical systems seamlessly as a third-party application, for example the Supplementary Materials (2. Demonstration of the operation procedure on laptop linked by email.mp4, and 3. Demonstration of the operation procedure on iPad via website.mp4). These features may play an assistant role to improve work patterns and extend the range of application of traditional medical image systems. For example, it can be applied to regional health, telemedicine, and medical imaging education at a low cost because there is no special cost of deploy and upgrade of software, network, and hardware on the client side. And more importantly, a teacher can vividly show the students the image characteristics of the disease anywhere; a clinician can intuitively introduce disease to the patients anywhere and ask experts' help anywhere. That may promote the development of mobile medical technology to a certain degree.

## Supplementary Material

1. Demonstration of the operation procedure on laptop via website.
2. Demonstration of the operation procedure on laptop linked by email.
3. Demonstration of the operation procedure on iPad via website.

## Figures and Tables

**Table 1:** Relational data storage structure of request behaviors from client and resource allocation on the server.

Table	Field		Note
*Base table*	DICOMs_ID	PRIMARY KEY, the unique identifier of a group of SMIs	To record the basic information of SMIs on the server side
	Storage path	The storage path of SMIs on the server side	

*Pipeline table*	pipeline_ID	PRIMARY KEY, the unique identifier of a pipeline for viewing a group of SMIs between the client and server	Based on *base table*, to record the connected relationship between the SMIs rendering in memory on the server and remote user on the client
	DICOMs_ID	FOREIGN KEY of *base table*, to declare which group of SMIs the client is viewing	
	Client	Client user	
	IP address	IP address of the committer	
	State	Whether the SMIs finished rendering in memory on the server? *Yes*, allow the client to next operation. *No*, prompt for waiting	

*Behavior table*	behavior_ID	PRIMARY KEY, the unique identifier of each behavior request event from the client	Based on *pipeline table*, to record operation requests from the client user and their process state
	pipeline_ID	FOREIGN KEY of *pipeline table*, to declare which pipeline on viewing and processing	
	Behavior type	Including *viewing angle*, *clipping,* or *color/opacity* transformation	
	Behavior-instruction	From the client, to transfer the parameters in terms of the corresponding behavior type, recorded in the form of quantization parameters	
	State	*Pending*, *processing,* or *finished*	

**Table 2:** Statistic results of the five-point scale concerning the interactive experience and imaging quality.

Focus	Subject		Votes from five-point scale				
			Very agree	Agree	Uncertainty	Disagree	Very disagree
*Interactive experience:* Mouse-based △ Gesture-based ▲	The operation of viewing and processing is accurate	△	3				
		▲	3				
	Simple and easy to use without training	△	2	1			
		▲	3				
	A good user experience similar to native application	△	2	1			
		▲	2	1			

Imaging quality	*Slave* model could meet demands of navigation		2	1			
	2D image from* Master* volume could provide high quality reconstruction result via utilizing the display capability of Internet terminals		3				

Others	Latency time for final high quality images		3				

**Table 3:** Average response time and amount of data transmission from the different behaviors and devices at 100-KBps.

Devices	First loading (average value)		Behavior request for final image (average value)		Idle status of bandwidth occupation (KBps)	Frames Per Second (FPS)
	Amount of data trans (KB)	Response time (seconds)	Amount of data trans (KB)	Response time (seconds)		
PC (3G net)	1085.44	11.23	47.10	1.12	0.23	48–60
Laptop (WIFI)	1064.96	10.51	46.08	0.89	0.31	48–60
iPad mini (WIFI)	1079.41	10.93	44.03	0.95	0.35	50–60
Smartphone (3G net)	1075.02	10.72	39.94	1.02	0.28	30–40
